# Therapeutic Outcome of Fluorescence Cystoscopy Guided Transurethral Resection in Patients with Non-Muscle Invasive Bladder Cancer: A Meta-Analysis of Randomized Controlled Trials

**DOI:** 10.1371/journal.pone.0074142

**Published:** 2013-09-13

**Authors:** Haichao Yuan, Jianguo Qiu, Liangren Liu, Shuo Zheng, Lu Yang, Zhenghua Liu, Chunxiao Pu, Jinhong Li, Qiang Wei, Ping Han

**Affiliations:** 1 Department of Urology, West China Hospital, Sichuan University, Guoxue Xiang #37, Chengdu, Sichuan, P. R. China; 2 Department of Hepatobiliary Pancreatic Surgery, West China Hospital, Sichuan University, Guoxue Xiang #37, Chengdu, Sichuan, P. R. China; Centro Nacional de Investigaciones Oncológicas (CNIO), Spain

## Abstract

**Objectives:**

To conduct a meta-analysis of randomized controlled trials (RCTs) to assess the therapeutic outcome of fluorescence cystoscopy (FC) guided transurethral resection (TUR) in non-muscle invasive bladder cancer (NMIBC).

**Materials and Methods:**

Relevant RCTs were identified from electronic database (MEDLINE, Embase and the Cochrane Library). The proceedings of relevant congress were also searched. The primary parameters were recurrence rate, the time to fist recurrence, recurrence free survival rate (RFS) and progression rate.

**Results:**

12 RCTs including 2258 patients, which were identified for analysis in our study. Our study showed that the FC group have lower recurrence rate than the white light cystoscopy (WLC) group with statistically significant difference (OR: 0.5; p<0.00001). The time of the FC group first recurrence delayed significantly 7.39 weeks than WLC group (MD: 7.39 weeks; p<0.0001). There was a statistically significant difference in favor of FC in RFS at 1 yr (HR: 0.69; p<0.00001) and 2 yrs (HR: 0.65; p=0.0004). However, the FC group cannot significantly reduce the rate of progression into muscle invasive bladder cancer compared with the WLC group (OR: 0.85; p=0.39).

**Conclusions:**

FC guided TUR was demonstrated to be an effective procedure for delaying recurrence of NMIBC. Unfortunately, FC guided TUR could not significantly decrease the rate of progression into muscle invasive bladder cancer.

## Introduction

Transurethral resection (TUR) with cystoscopy is the current main treatment for non-muscle-invasive bladder cancer (NMIBC), but residual tumour was found in 30%–44% patients after initial treatment [1]. This rate could exceed 70% for high-grade tumours such as carcinoma in situ (CIS) [2]. Furthermore, the probability for recurrence of NMIBC at 1 and 5 years had been reported as 15%-61% and 31%–78%, respectively, whereas the rates for progression at 1 and 5 years had been reported as <1%–17% and <1%-45%, respectively [3]. Therefore, tumour recurrence in patients with NMIBC was a common problem.

Residual tumours that were undetected or overlooked during initial TUR may contribute to recurrence. White light cystoscopy (WLC) was considered the current standard method for detecting tumours during TUR, however, its sensitivity and specificity was not entirely satisfactory [4]. Small papillary bladder tumours and CIS were very difficult to detect using WLC; therefore, this method was associated with a potential risk of recurrence [5]. Therefore, there was an urgent need for improving the sensitivity of cystoscopy.

Fluorescence cystoscopy (FC) had been introduced for NMIBC diagnosis and treatment [6]. Using this method, photoactive porphyrins such as 5-aminolaevulinic acid (5-ALA) or hexylaminolevulinate (HAL) were used to instill into the bladder and emitted red fluorescence under blue light. This method, also known as photodynamic diagnosis, had been studied extensively in recent years.

Several studies [7,8] had demonstrated that FC was more sensitive than WLC in detecting small papillary bladder tumours and CIS, thus improving tumour detection rates and decreasing residual tumour rates. Furthermore, no significant adverse effects related to the use of this method had been reported till date. FC had received approval for use in the detection of bladder cancer in several countries. However, debate continues about the applicability because of cost-effectiveness of FC, it may be used only for suspected high-grade tumours according to the guidelines proposed by the European Association of Urology (EAU) [9]. No overall consensus has been reached in Europe or other regions concerning the use of this technique in all patients or only selected patients.

Three previously conducted meta-analyses demonstrated the superiority of FC over WLC in the detection of bladder tumours, especially CIS [7,8,10]. According to the results of these studies, complete resection was more often achieved with FC, thus demonstrating the diagnostic accuracy of FC in patients with NMIBC. However, insufficient data concerning the therapeutic outcome of FC in patients with NMIBC were available. Evidence regarding tumour recurrence and progression was still lacking.

This study aimed to conduct a meta-analysis of evidence from randomized controlled trials (RCTs) to assess the therapeutic outcome of FC guided TUR in patients with NMIBC.

## Materials and Methods

### Study selection

In accordance with a pre-specified study protocol, an electronic database search of Medline, Embase and the Cochrane database was systematically undertaken to identify studies conducted between 1996 and October 2012. The search terms belonged to the Medical Subject Headings database and included fluorescence cystoscopy, photodynamic diagnoses, bladder cancer/tumour, white light cystoscopy, randomized controlled trial. These terms were searched individually and in combination. The proceedings of the American Society of Clinical Oncology, the European Association of Urology and the American Urological Association were also manually searched. Moreover, the reference lists of all included studies were scanned to identify additional potentially relevant studies. Searches were restricted to English language publications. The search was independently conducted by two authors (HCY, JGQ). Any discrepancies were resolved in consultation with the third author (PH).

### Inclusion criteria

RCTs that assessed the clinical efficacy of FC and compared it with that of WLC in patients with suspected or proven NMIBC were included. These studies included at least one outcome of interest to the current study. When two or more studies reported on a group of patients at the same institution during an overlapping time period, the study with the longest follow-up period was included.

### Outcomes of interest

The following outcome data were extracted from the included studies:

1 Recurrence rate: the number of bladder cancer recurrences after initial TUR
2 Recurrence-free survival rate at 1 year
3 Recurrence-free survival rate at 2 years
4 Time to first recurrence, defined as the time until bladder cancer recurrence after initial TUR
5 Progression rate, defined as the number of patients with disease progression into muscle invasive bladder cancer during the follow-up period.

### Study quality assessment

The quality of the literature was assessed separately by two authors (HCY and JGQ) using the 6 items of the Jadad scale score [11]. Scores of 0 to 8 were allocated to each study. Studies with scores of 5 points or more were defined as high-quality studies. Those with scores of 3 or 4 were designated as moderate quality, and those that scored 2 points or less were of low quality.

### Data extraction and statistical analysis

The meta-analysis was performed in line with the recommendations of the Cochrane Collaboration [12]. Two reviewers (HCY and JGQ) reviewed the selected studies and independently extracted the following information: study design, year of publication, study population characteristics and relevant outcome data. Any discrepancy was resolved in consultation with the third author (PH). Statistical analysis of dichotomous variables was performed using the odds ratio (OR) as the summary statistic, while continuous variables were analyzed using the weighted mean difference (MD). For both variables, 95% confidence intervals (CI) were reported. When the outcome data involved comparison of two survival curves such as those for recurrence-free survival (RFS), the log hazard ratio statistic was used. In some studies, only the mean and p-values for the time of first recurrence, the p-value derived in log-rank tests for RFS and the events observed in each group were reported, and therefore the standard deviation (SD) and hazard ratio (HR) were estimated using the statistical methods^12^.

Heterogeneity was assessed using the I^2^ statistic. The Mantel–Haenszel chi-squared test for heterogeneity was performed. I^2^ values of <25% were defined as low heterogeneity, those between 25% and 50% as moderate heterogeneity and those >50% as high heterogeneity. In case of lack of heterogeneity, fixed-effects model was used for the meta-analysis, or else random-effects model was used. When data were reliable and sufficient, subgroup analysis was introduced by grouping the trials to determine possible heterogeneity and bias. For these tests, a p-value of <0.05 was considered statistically significant. The analysis was conducted using Review Manager Version RevMan 5.0.

## Results

### Search results and reporting quality

**Figure 1:**
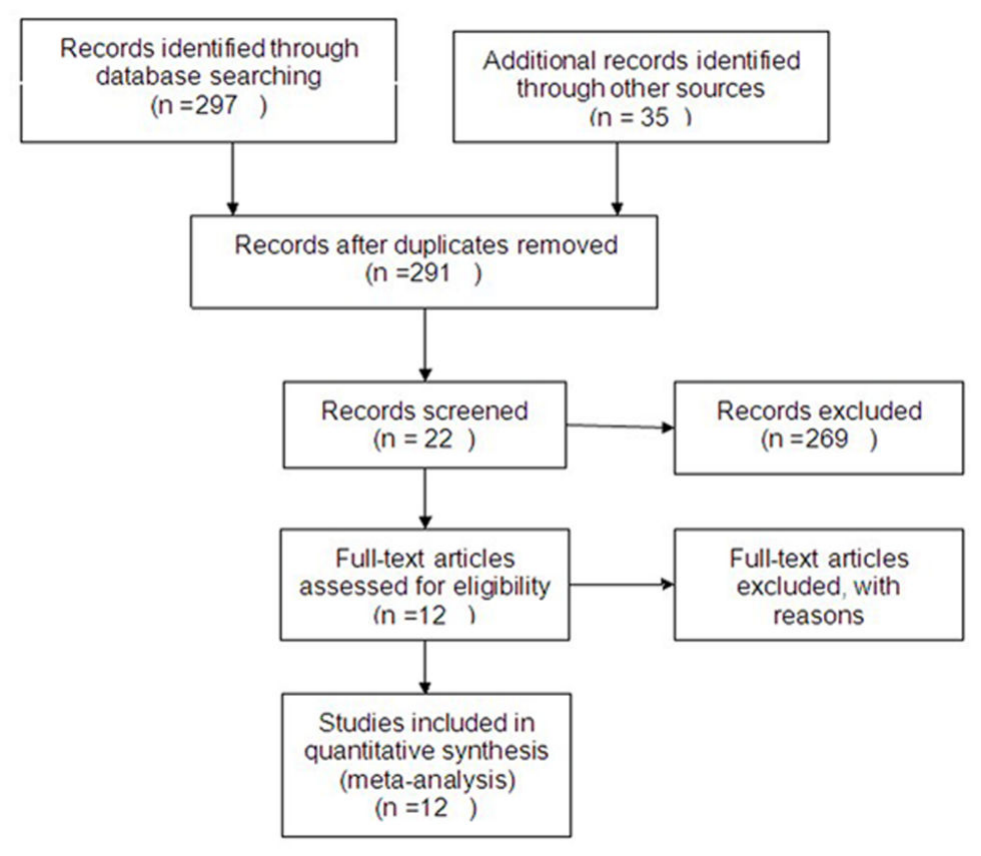
Flow diagram of evidence acquisition.

Using the search strategy described previously, after study assessment there were 12 RCTs [13-24] for analysis in this review. Of these RCTs, two [19,20] were from the same authors or institution, but the extracted outcome data were different. These studies included 2258 patients in whom FC or WLC had been performed for NMIBC. The FC group was divided into two subgroups: the 5-ALA group and HAL group. Baseline information was comparable between the FC and WLC groups. The baseline characteristics and quality assessment of the included studies are summarized in Table 1. Flow diagram of evidence acquisition is illustrated in Figure 1.

**Table 1:** Baseline characteristics and quality assessment of the Included Studies.

Study	Age(year)	Patient sex (M/F)	FA	Jadad scale score (6 Items)	Study type	Cases	follow-up (months)
Riedl CR 2001	-	-	5-ALA	6	RCT	51/51	-
Kriegmair M 2002	69.3/69.6	53:12/45:19	5-ALA	5	RCT	65/64	-
Filbeck T 2002	70/68	-	5-ALA	6	RCT	88/103	21.2/20.5
Babjuk M 2005	69.8/67.9	39:23/43:17	5-ALA	5	RCT	60/62	20.7/22.4
Schumacher CM 2010	68.9/70.1	104:34/103:38	5-ALA	6	RCT	138/141	12/12
Stenzl A 2011	-	-	5-ALA	7	RCT	183/187	12/12
Stenzl A 2010	68/69.6	212:59/223:57	HAL	5	RCT	271/280	12/12
Grossman HB 2012							
Dragoescu.O 2011	62/58	16:6/18:4	HAL	5	RCT	22/22	9/9
Geavlete B 2012	-	-	HAL	7	RCT	114/125	24/24
Hermann GG 2011	69/71	58:19/51:17	HAL	5	RCT	77/68	12/12
Karaolides T 2012	63.8/66.2	40:5/33:8	HAL	6	RCT	45/41	18/18

M = male; F = female; 5-ALA = 5-aminolevulinic acid; HAL = hexylaminolaevulinic acid; CIS = carcinoma in situ; FA = fluorescence agent; n = number of patients; RCT = randomized controlled trials.

### Recurrence rate

**Figure 2:**
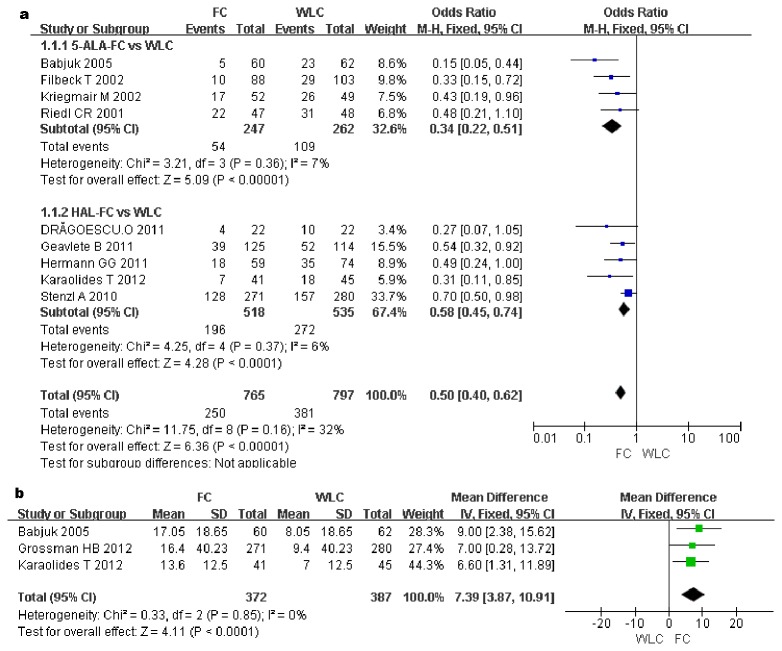
Forest plot of FC vs. WLC for recurrence rate (a). Forest plot of FC vs. WLC for the time to first recurrence (week) (b).

The recurrence rate was measured in nine studies including 1562 patients. The recurrence rate was significantly lower in the FC group than in the WLC group (OR, 0.5; 95%CI, 0.4–0.62; p<0.00001). Subgroup analysis revealed a statistically significant difference between the WLC group and the 5-ALA group (OR, 0.34; 95%CI, 0.22–0.51; p<0.00001) and between the WLC group and the HAL group (OR, 0.58; 95%CI, 0.45–0.74; p<0.0001) (Figure 2a).

### The Time to first recurrence (weeks)

The time to first recurrence was reported in three RCTs involving 759 patients. The pooled estimates of these studies showed a statistically significant difference between the FC and WLC groups (MD, 7.39 week; 95%CI, 3.87–10.91; p<0.0001). This result indicates that the time of first recurrence in the FC group was delayed significantly (7.39 weeks) compared with that in the WLC group (Figure 2b).

### RFS rates at 1 and 2 years

**Figure 3:**
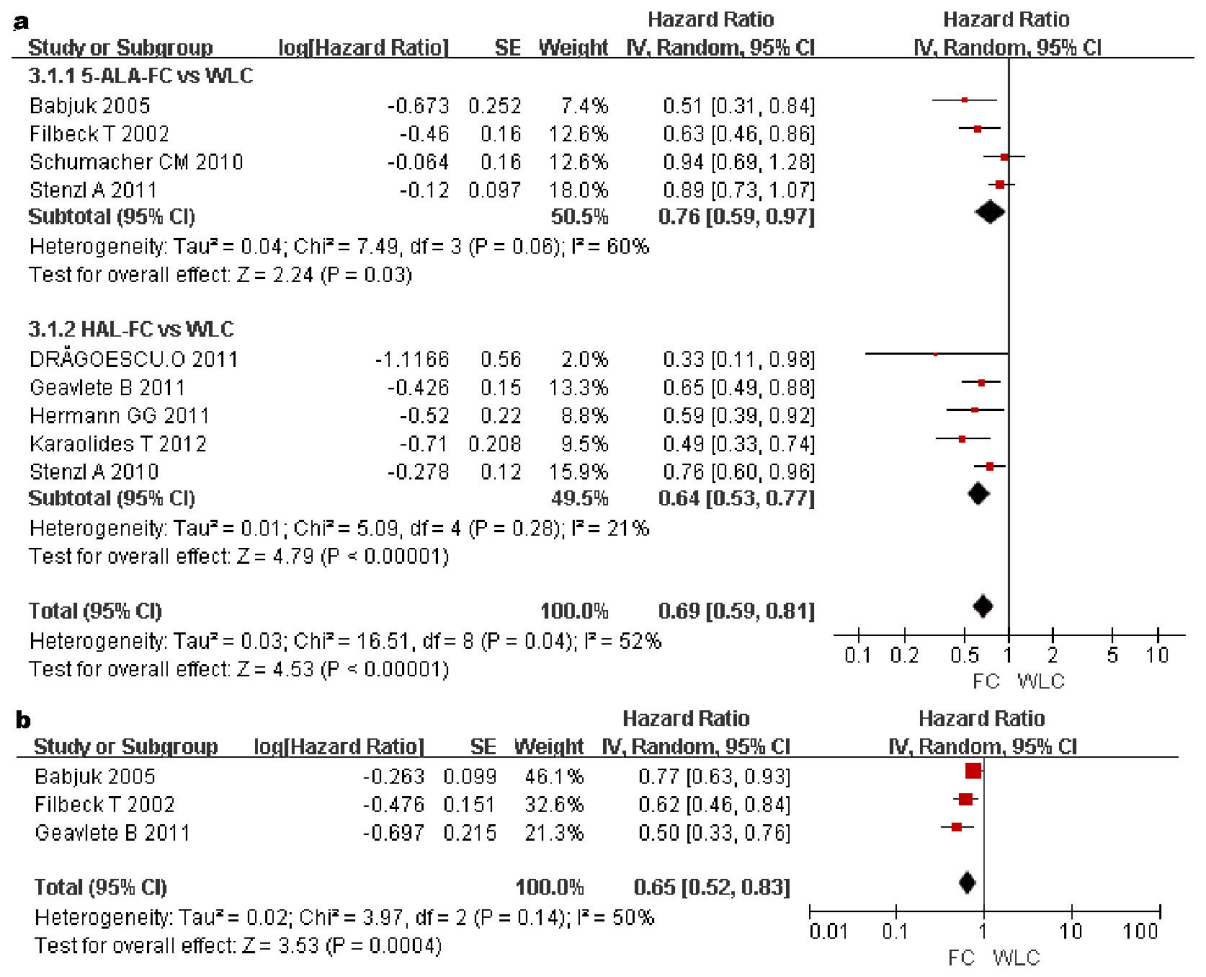
Forest plot of FC vs. WLC for RFS rate at 1 year (a). Forest plot of FC vs. WLC for RFS rate at 2 years (b).

Nine studies involving 2027 patients and three studies involving 552 patients reported RFS rates at 1 and 2 years. In the pooled estimates, a statistically significant difference in favour of FC was observed at 1 (HR, 0.69; 95%CI, 0.59–0.81; p<0.00001) and 2 years (HR, 0.65; 95%CI, 0.52–0.83; p=0.0004) (Figure 3a and 3b). Subgroup analysis also detected a statistically significant difference between the WLC and 5-ALA group at 1 year (HR, 0.76; 95%CI, 0.59–0.97; p = 0.03) and between the WLC and HAL groups at 1 year (HR, 0.64; 95%CI, 0.53–0.77; p<0.00001) (Figure 3a). Therefore, RFS rates at 1 and 2 years were higher in the FC group than in the WLC group.

### Progression rate

**Figure 4:**
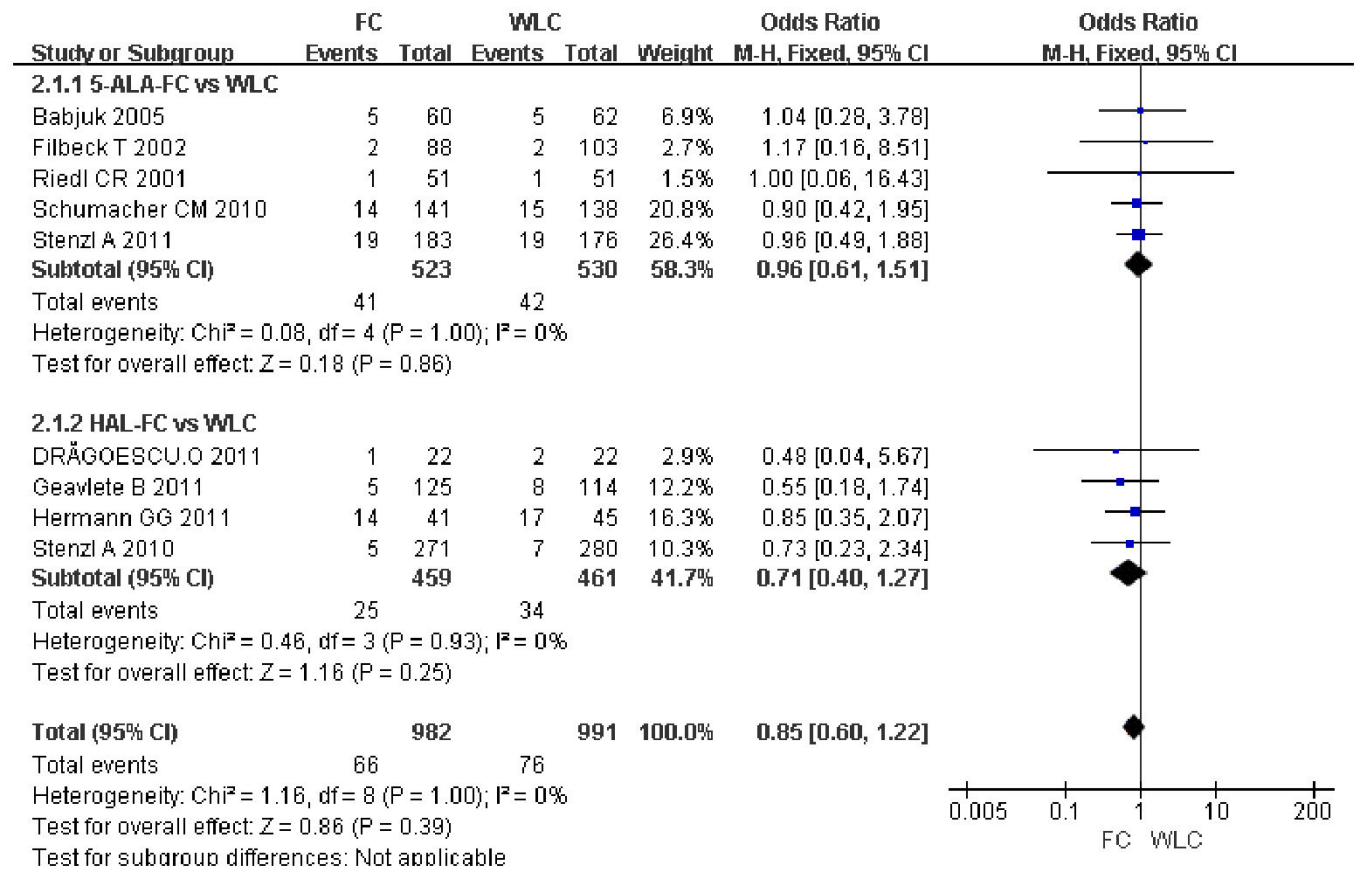
Forest plot of FC vs. WLC for progression rate.

Nine studies including 1973 patients reported on the rate of progression. A meta-analysis of these studies showed that no significant difference was observed in the rate of progression into muscle invasive bladder cancer between the FC group and the WLC group (OR, 0.85; 95% CI, 0.6–1.22; p = 0.39). Subgroup analysis found no significant difference in the rate of progression between the WLC and 5-ALA group (OR, 0.96; 95%CI, 0.61–1.51; p = 0.86) and between the WLC and HAL group (OR, 0.71; 95% CI, 0.40–1.27; p= 0.25) (Figure 4).

## Discussion

The benefit for FC which could detect more bladder tumours and reduce residual tumours had been proved previously by another systematic review and meta-analysis [7,8]. The findings in these studies indicated that this technique may result in more complete tumour resection and decreased recurrence rates. This conclusion was also supported by the results of the current study that showed that the recurrence rate was significantly lower, the time to first recurrence after initial TUR was prolonged and RFS rate at 1 and 2 years was improved when FC-guided TUR was used than when WLC was used. However, there was no significant decrease in the rate of progression into muscle invasive bladder cancer in patients who underwent FC.

Three previously meta-analyses showed the superiority of FC over WLC in the detection of bladder tumours, especially CIS [7,8,10], which demonstrate the diagnostic accuracy of FC in patients with NMIBC. However, the objective of our meta-analysis was to evaluate the effect of FC in patients with NMIBC. Another meta-analysis of the use of FC in patients with NMIBC, that of Shen et al [25], found no significant difference in tumour detection rate and RFS between the FC and WLC groups. Their study found no advantage of FC over conventional WLC in terms of diagnostic accuracy and therapeutic outcome. In their study, 14 RCTs were included. However, one of these studies was a retrospective study, and 3 had been published repeatedly. These factors may have caused a publication bias. The heterogeneity of the pooled results was also very high and the method of data extraction for the RFS rate was unreasonable. Therefore, these results should be interpreted with caution as a result of the potential risk of bias.

We also found that differences in RFS rates between patients in the WLC group and those in the FC subgroups were not coinciding. Babjuk et al [16], Geavlete et al [22] and Karaolides et al [24] reported no statistically significant difference in RFS at 1 year between the FC and WLC groups when solitary tumours were treated (p = 0.74, 0.064 and 0.352, respectively); however, a statistically significant difference in RFS was observed between the FC and WLC groups when multifocal tumours were treated (p = 0.001, 0.001 and <0.001, respectively). The superiority of FC was particularly obvious in the intermediate-risk (p = 0.02) and high-risk (p = 0.05) groups, but this advantage did not achieve statistical significance in the low-risk group (p = 0.25) [15]. Data for these subgroups were insufficient for inclusion in the current meta-analysis.

The results of this study also showed that although the recurrence rate was significantly decreased and time to recurrence was longer in patients who underwent FC, these improvements did not translate into a decrease in the rate of progression into muscle invasive bladder cancer. We considered that one factor underlying this finding may be that patients with high-risk tumours should be treated with adjuvant therapy such as bacillus Calmette–Guérin instillation, regardless of whether CIS was easily found. Another factor may be that the sensitivity of FC, especially for CIS, is as yet uncertain [26]. Some studies have reported that FC may result in a higher incidence of false-positive results compared with WLC. However, with increased experience with and technical improvements in FC, false-positive rates are dropping. One study showed that only a 1% difference in the rate of false positives is existed between the FC and WLC groups [27].

All meta-analyses are inherently limited by the quality of the primary studies. Fortunately, all included studies in the current meta-analysis were RCTs, and most were of high quality. However, the statistical data in some included studies were incomplete, detailed data could not be acquired by contacting the relevant authors, so these data were lost. In addition, some parameters important to this meta-analysis were only measured in certain studies. These factors may be a possible source of bias. Subgroup analysis such as solitary vs multifocal tumors or low vs high-risk tumor could not be conducted, which may be reveal more useful information, but few study perform a relative subgroup study, we cannot extract sufficient data to conduct the subgroup analysis. This was another limitation of the current study. Moreover, some studies [13-19] used 5-ALA as a photoactive porphyrin, while others [19-24] used HAL. The factor may have introduced heterogeneity in the present results. However, the heterogeneity of all of the pooled estimates in this review was acceptable. Therefore, the results of this study are realistic because they were based on available data and high-quality RCTs.

Bladder cancer is an expensive cancer to treat. The lifetime cost per patient has been estimated at up to $200,000, which puts an enormous economic stress on the medical system [28]. Most costs are incurred in association with NMIBC, which has a life-long tendency to recur in most cases and requires repeated endoscopic surgery. FC is gradually becoming accepted as a useful tool for the diagnosis and management of NMIBC. Furthermore, the EAU guidelines [9] also provide recommendations for the use of FC in patients with NMIBC.

The results of this study showed that the use of FC during initial TUR-BT can facilitate more complete tumour resection, resulting in a decreased recurrence rate, prolonged the time of first recurrence and improved RFS at 1 and 2 years. Therefore, FC can change the therapeutic strategy for patients with NMIBC, longer intervals to follow-up with cystoscopy, fewer TUR-BT procedures and less adjuvant therapy will become possible for the treatment of NMIBC in patients who have undergone FC. Burger et al [29] reported that a single extra cost of €135 for FC resulted in savings of €187 per patient per year over a follow-up period of more than 7 years among patients who underwent FC. Therefore, improved prognostic outcome of bladder cancer in patients who undergo FC can decrease long-term costs of care and follow-up, despite the additional costs for fluorescent agents and learning of this modified technique [29,30]. This finding suggested that the use of FC could result in savings. Further studies evaluating the use of FC from an economic point of view are needed. However, FC is likely to have a positive effect not only on prognosis but also on patient quality of life [30].

Recently, flexible cystoscopy was widely used in detecting bladder tumors and in the follow-up of bladder cancer patients [31,32]. Some studies reported that PDD-guided flexible cystoscopy could identifies smaller, more papillary tumors or flat CIS lesions in anaesthetized patients in the operating room than white light cystoscopy and rigid cystoscopes [33,34]. Hermann GG et al [35] reported that PDD-guided flexible cystoscopy can be performed in an outpatients department setting and that simultaneous biopsies are able to give a reliable histological diagnosis of bladder cancer. If CIS and high-risk non-muscle-invasive bladder tumours were followed in the outpatients department with flexible cystoscopes under local anaesthesia instead of anaesthesia in the operating room, many resources could be spared for patients and the health system. But Flex biopsies are not sufficiently reliable to identify muscle invasive bladder cancer because of a low presentation of muscularis propria in the biopsies and thus a risk of overlooking, therefore, patients with high-grade disease should be referred to the operating room for cystoscopy and biopsy. However, further research should be performed to assess the effect of PDD-guided flexible cystoscopy.

Further research focusing on larger, multicentre RCTs and comparing subgroups of patients such as solitary vs multifocal tumors or low vs high-risk tumor with NMIBC are required. Such research may provide urologists with more comprehensive and detailed recommendations concerning the management of patients with NMIBC.

## Conclusions

Compared with WLC, FC guided TUR could significantly decrease recurrence rates, prolong the time to first recurrence after initial TUR and improve RFS at 1 and 2 years. Therefore FC was demonstrated to be an effective procedure for delaying recurrence of NMIBC. The benefits of FC may be result in savings and decrease the burden of the cost of caring for patients with NMIBC on the health care economy. Unfortunately, FC guided TUR could not significantly decrease the rate of progression into muscle invasive bladder cancer. Further studies are required to explore possible reasons.
